# Remote Symptom Monitoring Using Patient-Reported Outcomes in Patients With Chronic Kidney Disease: Process Evaluation of a Randomized Controlled Trial

**DOI:** 10.2196/48173

**Published:** 2024-04-24

**Authors:** Birgith Engelst Grove, Annette de Thurah, Per Ivarsen, Ann Katrine Kvisgaard, Niels Henrik Hjollund, Regine Grytnes, Liv Marit Valen Schougaard

**Affiliations:** 1 AmbuFlex, Centre for Patient-Reported Outcomes Gødstrup Hospital Herning Denmark; 2 Department of Clinical Medicine Aarhus University Aarhus Denmark; 3 Department of Rheumatology Aarhus University Hospital Aarhus Denmark; 4 Department of Renal Medicine Aarhus University Hospital Aarhus Denmark; 5 Department of Clinical Epidemiology Aarhus University Aarhus Denmark

**Keywords:** chronic kidney disease, pragmatic randomized controlled trial, process evaluation, patient-reported outcome measures, remote monitoring, monitoring, patient-reported outcome, chronic kidney, intervention

## Abstract

**Background:**

In Denmark, outpatient follow-up for patients with chronic kidney disease (CKD) is changing from in-hospital visits toward more remote health care delivery. The nonuse of remote patient-reported outcomes (PROs) is a well-known challenge, and it can be difficult to explain which mechanisms of interventions influence the outcome. Process evaluation may, therefore, be used to answer important questions on how and why interventions work, aiming to enhance the implications for clinical practice.

**Objective:**

This study aimed to provide insight into the intervention process by evaluating (1) the representativity of the study population, (2) patient and physician use patterns, (3) patient adherence to the intervention, and (4) clinical engagement.

**Methods:**

A process evaluation determining the reach, dose, fidelity, and clinical engagement was carried out, alongside a multicenter randomized controlled trial (RCT). We developed and implemented an intervention using PRO measures to monitor outpatients remotely. Data were collected for the PRO intervention arms in the RCT from 4 sources: (1) PRO data from the participants to determine personal factors, (2) the web-based PRO system to identify key usage intervention patterns, (3) medical records to identify clinical factors relating to the use of the intervention, and (4) semistructured interviews conducted with involved physicians.

**Results:**

Of the 320 patients invited, 152 (47.5%) accepted to participate. The study population reflected the target population. The mean adherence rate to the PRO intervention arms was 82% (95% CI 76-87). The questionnaire response rate was 539/544 (99.1%). A minority of 13 (12.9%) of 101 patients needed assistance to complete study procedures. Physicians assessed 477/539 (88.5%) of the questionnaires. Contact was established in 417/539 (77.4%) of the cases, and 122/539 (22.6%) of the patients did not have contact. Physicians initiated 288/417 (69.1%) and patients requested 129/417 (30.9%) of all the contacts. The primary causes of contact were clinical data (242/417, 58%), PRO data (92/417, 22.1%), and medication concerns and precautionary reasons (83/417, 19.9%). Physicians found the use of PRO measures in remote follow-up beneficial for assessing the patient’s health. The inclusion of self-reported clinical data in the questionnaire motivated physicians to assess patient responses. However, some barriers were emphasized, such as loss of a personal relationship with the patient and the risk of missing important symptoms in the absence of a face-to-face assessment.

**Conclusions:**

This study demonstrates the importance and practical use of remote monitoring among patients with CKD. Overall, the intervention was implemented as intended. We observed high patient adherence rates, and the physicians managed most questionnaires. Some physicians worried that distance from the patients made it unfeasible to use their “clinical glance,” posing a potential risk of overlooking crucial patients‘ symptoms. These findings underscore key considerations for the implementation of remote follow-up. Introducing a hybrid approach combining remote and face-to-face consultations may address these concerns.

**Trial Registration:**

ClinicalTrials.gov NCT03847766; https://clinicaltrials.gov/study/NCT03847766

## Introduction

Lifestyle and a growing elderly population in Denmark have increased the number of patients with chronic diseases to approximately 1 million in a population of 6 million, with growing health care expenditure as a consequence [1]. To improve efficacy, new ways of delivering health care to people with chronic kidney disease (CKD) have been posited, such as remote monitoring, which is defined as using technology to monitor patients at a distance [2,3]. One way of remotely monitoring patients is to collect information about their symptoms by obtaining repetitive patient-reported outcomes (PROs) while the patients are at home. This allows for frequent capture of important disease-specific outcomes and also allows clinicians direct access to the patients’ health status [4,5]. PROs are measures of a patient’s health conveyed directly by the patient, without interpretation by a clinician or anyone else [6], often gathered from electronic questionnaires. Remote monitoring using PROs is increasingly applied among patients with chronic conditions in Denmark [4,7]. However, several personal and external factors may affect a person’s ability to engage with and use digital health interventions [8,9]. Prior studies have shown sociodemographic and economic inequality in patients who attend [10,11] and adhere to PRO-based remote follow-up [12].

A pragmatic randomized controlled trial (RCT) named PROKID (PRO measures in Kidney care) is currently investigating whether PRO-based remote follow-up is a safe and effective alternative to health care delivery to patients with CKD [7]. The primary outcome in the PROKID trial is renal function measured using the estimated glomerular filtration rate (eGFR) as this is the single-most accurate measurement for CKD progression [13] and thereby indicates the level of safety in remote monitoring. The effectiveness of remote monitoring is measured using clinical data, resource use, and PROs, such as the quality of life and illness perception [14]; also see Grove et al (unpublished data, 2024). PRO-based interventions in other patient populations have shown the ability to (1) reduce the number of outpatient visits [15,16], (2) improve communication between health care professionals and patients [17-20], and (3) offer patients a more comprehensive understanding and greater management of their condition [21-23]. However, several knowledge gaps regarding the use and acceptability of remote PRO interventions are still present. Trials are vulnerable to various biases that may undermine external validity [24]. Evidence suggests that often, this risk of bias is related to patients not using the technology as intended [25]. Thus, examining the quality (fidelity) and quantity (dose) of what was implemented in practice and the extent to which the intervention reached its intended users is essential when evaluating trials [26]. The degree to which patients engage with and use the intervention as intended is a crucial component in evaluations [26]. The nonuse of remote PRO is a well-known challenge [16,27] and may blur the overall interpretation of using PRO as the basis for remote follow-up. However, little is known about the components that might enhance patient and clinical engagement and thus might have the most significant impact in terms of use and adherence to the intervention. The PROKID trial [14] cannot explain which mechanisms of the intervention have an influence on the outcome. Process evaluation may therefore be used to answer important questions on how and why interventions work, aiming to enhance the implications for clinical practice [26].

Thus, we aimed to conduct a process evaluation to obtain insights into the PROKID intervention’s working mechanisms by evaluating (1) the representativity of the study population, (2) patient and physician use patterns, (3) patient adherence to the intervention, and (4) clinical engagement.

## Methods

### Study Setting

**Figure 1:**
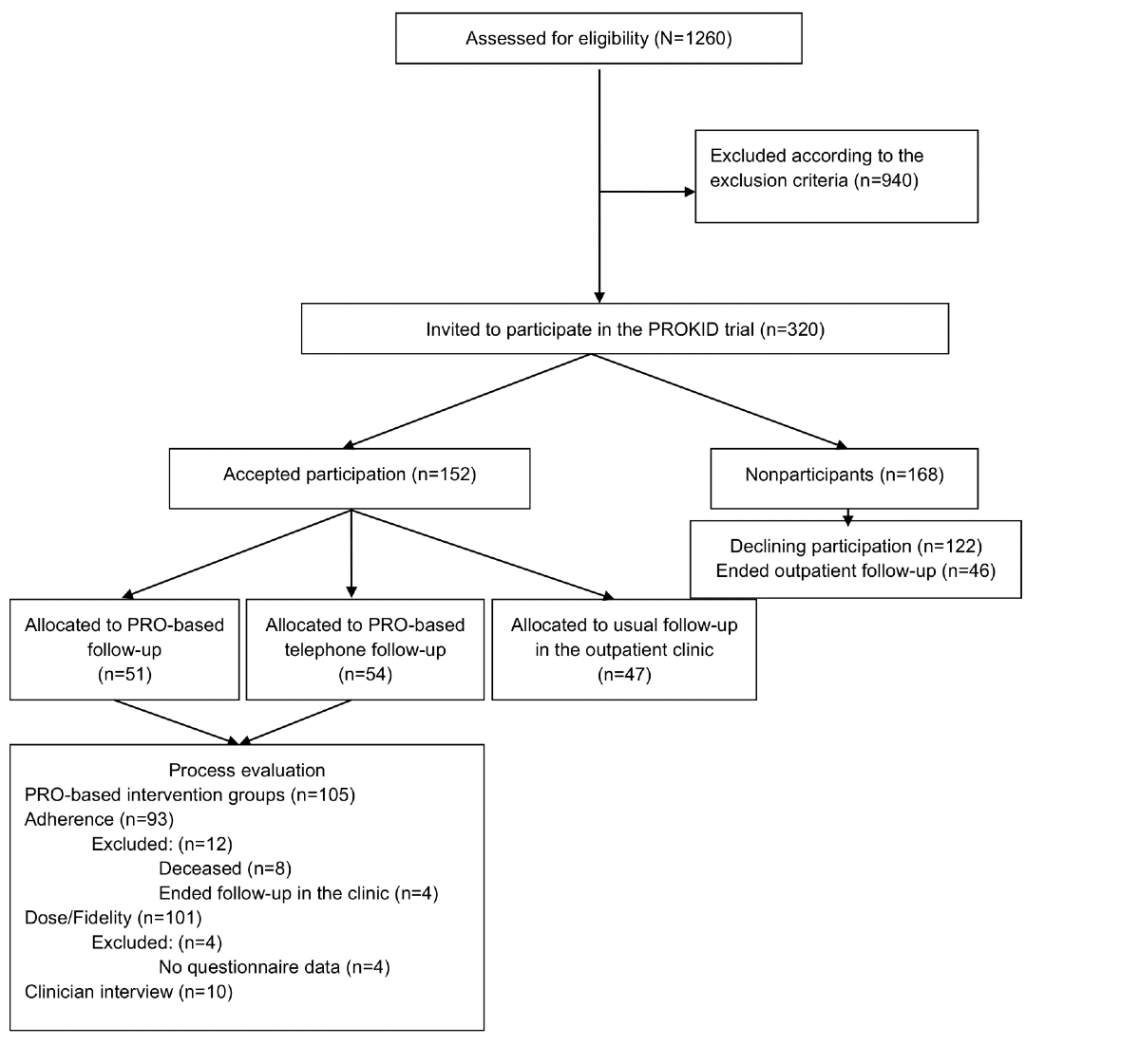
Recruitment of patients with CKD from Aarhus University Hospital, Gødstrup Hospital, and Viborg Regional Hospital to the PROKID trial and process evaluation from January 2019 to August 2021. CKD: chronic kidney disease; PROKID: PRO measures in Kidney care.

In 2019, the PROKID trial, a Danish multicenter RCT, was initiated to evaluate the noninferiority of PRO-based remote follow-up compared to usual outpatient clinic visits in managing the decline in renal function and maintaining patients’ quality of life [7]. The intervention was implemented in a real-life setting, and clinicians and patients were involved in the development process. We provided short oral seminars for the staff in the outpatient clinics, where the intervention was discussed and refined. We invited patients to provide input into the design and the patient information sheets of the intervention. Participants were eligible for the trial if they attended follow-up from January 2019 to August 2021 at a renal outpatient clinic in Central Denmark Region and had a renal function of eGFR≤40 mL/minute. In addition, participants had to be ≥18 years old and able to complete a questionnaire. Newly referred outpatients were randomized into the following 3 groups: (1) PRO-based follow-up, (2) PRO-based telephone follow-up, or (3) usual outpatient follow-up (Figure 1).

**Figure 2:**
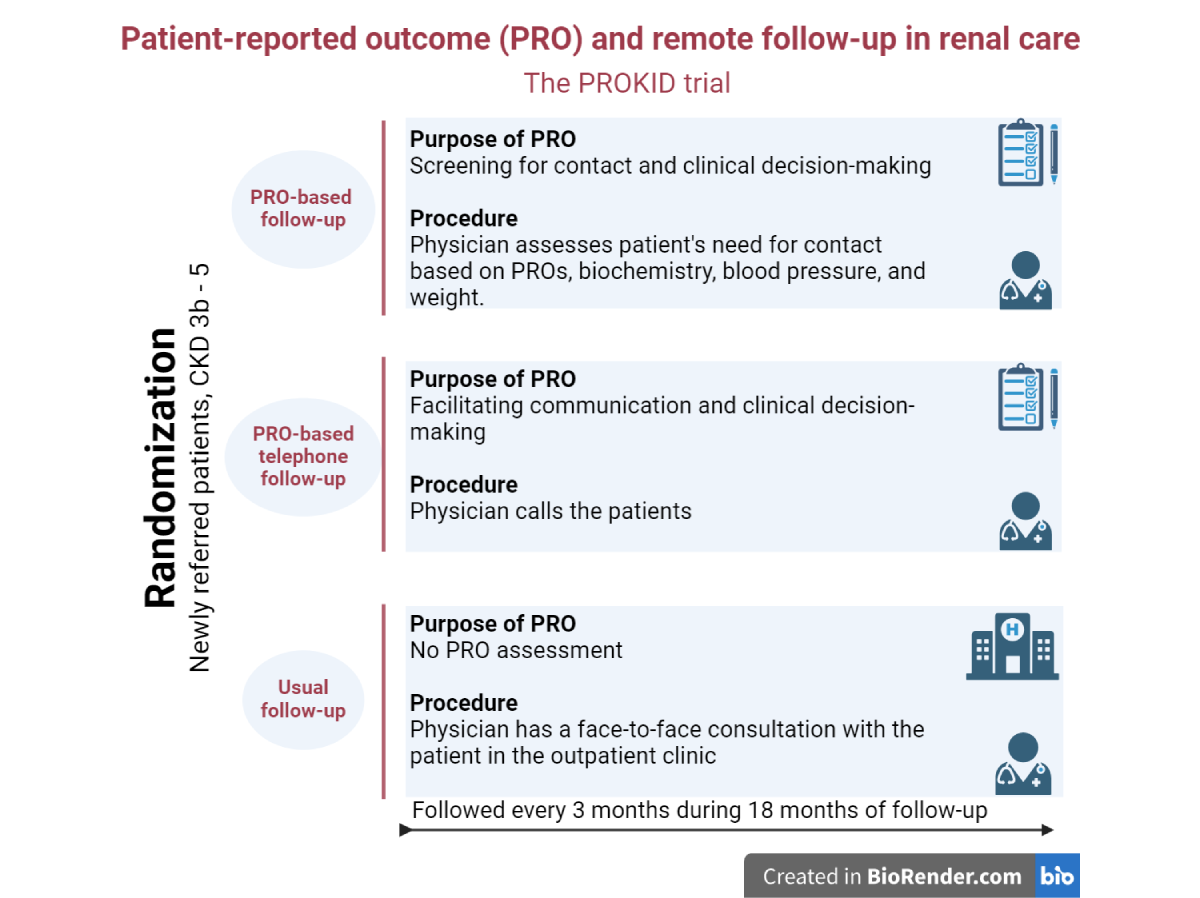
Overview of the intervention groups in the PROKID trial including patients with CKD at Aarhus University Hospital, Gødstrup Hospital, and Viborg Regional Hospital from January 2019 to August 2021. CKD: chronic kidney disease; PROKID: PRO measures in Kidney care.

In the intervention arms, each patient participated in 6 PRO consultations (when PROs were used in a consultation) during 18 months of follow-up. Prior to each PRO consultation, patients needed to undergo blood tests, measure their blood pressure and weight, and complete a disease-specific questionnaire. The questionnaires were sent to patients 7 days ahead of each consultation, and in the case of nonresponse, they were sent reminders on the fourth day and the day just prior to the consultation. The questionnaire included information about blood pressure, weight, self-rated health, renal-specific symptoms, and a free-text box [28]. The physicians accessed the patients’ PRO responses through a graphical overview embedded in the electronic health record system [5]. In the PRO-based follow-up intervention group, physicians had to approve each patient’s responses, manage them according to a color-coded algorithm, and determine whether the patient needed contact. A clinical expert group has assigned a color to each item response according to the severity of the symptom, as previously described: red, yellow, or green [14,28]. Each patient’s need for contact was evaluated based on their PRO responses and other clinical data, such as blood samples and blood pressure. Therefore, it was up to the physicians to decide whether contact with the patient was needed. This applied not only to instances where the color code was red or yellow but also to instances in which the questionnaire displayed a green color code. In the PRO-based telephone follow-up group, the physicians had to approve each patient’s response, call the patient, and document the conversation as conducted. Figure 2 outlines the groups, the content, and the purpose of using PRO.

### Study Design

The process evaluation was an integral component of the PROKID trial and focused on adding information not provided in the trial and clarifying how the intervention was received in practice to increase accuracy in the trial results. The design and methods of the PROKID trial are described elsewhere [7]. The design of this evaluation was influenced by Steckler and Linnan’s [26] process evaluation framework, building on 4 themes: reach, dose, fidelity, and clinical engagement. This study used qualitative and quantitative methods to answer the research questions. The study was reported in accordance with Consolidated Standards of Reporting Trials of Electronic and Mobile Health Applications and Online Telehealth (CONSORT-EHEALTH) guidelines [29].

### Participants

We obtained data from patients attending outpatient follow-up at the renal outpatient clinics at Aarhus University Hospital, Gødstrup Hospital, and Viborg Regional Hospital in Central Denmark Region. In the quantitative phase of the process evaluation, aimed to identify the reach of the intervention, the sample consisted of 320 patients, all eligible for randomization. The PROKID trial recruited 152 (47.5%) patients, and the 105 (69.1%) patients allocated to the PRO intervention groups informed the dose and fidelity of the trial. The qualitative phase of the process evaluation consisted of semistructured interviews with physicians (N=10) involved in the PROKID trial (Figure 1). Physicians were purposefully sampled from all involved outpatient clinics. Of the 10 physicians, 5 (50%) were female, 6 (60%) were senior consultants, and 4 (40%) were consultants, and their ages ranged from 37 to 67 years. Most of them had under 2 years of experience in using PRO in clinical practice.

### Process Evaluation Components

The research team identified core process questions for the evaluation stages. These related to describing the quantity and quality of what was delivered, covered by the following 4 components: reach, fidelity, dose, and clinical engagement [26]. An overview of the evaluation components and methods is outlined in Table 1.

**Table 1:** Research questions and key components in the process evaluation following the PROKID^a^ trial, Central Denmark Region.

Domain	Research questions	Applied method	Data source
Reach	Who receives the intervention, and is the sample representative? Why do patients disagree to participate?	Quantitative personal and clinical profiling of participants and nonparticipants Patient-reported reasons for nonparticipation	Hospital Business Intelligence Register AmbuFlex database Questionnaire data/PROs^b^
Dose	Who adheres to the intervention? To what extent have the patients received and engaged in the intervention?	Quantitative personal and clinical profiling in the level of adherence Number of distributed questionnaires and patient responses Number of item responses Time spent completing the questionnaire Color-coded algorithm Paper/web distribution Number of reminders	Hospital Business Intelligence Register AmbuFlex database REDCap^c^ database
Fidelity	Is the intervention delivered as intended? Do the physicians incorporate the patients’ PRO responses in the consultation?	Number of clinics and physicians using the system Physician assessments of patient responses (contact/no contact) Number of physicians involved in each patient pathway	AmbuFlex database
Clinical engagement	How do the physicians perceive the intervention in clinical practice?	Individual semistructured interviews	Involved physicians from all centers

^a^PROKID: PRO measures in Kidney care.

^b^PRO: patient-reported outcome.

^c^REDCap: Research Electronic Data Capture.

### Reach

Reach was measured by the degree to which the intended target population participated in the intervention. Participation was defined as patients being eligible and willing to participate in the PROKID trial. Participants and nonparticipants were compared according to demographic information and PROs, measured using questionnaires and clinical data collected before entrance to the trial. All patients who declined to participate were encouraged to provide a reason for refusal. A complete overview of the data sources and the PROs and clinical outcomes is presented in Table S1 in Multimedia Appendix 1.

### Dose

The assessment encompassed measuring the quantity of the intervention components given to the patients, as well as gauging the extent to which patients engaged in and adhered to the intervention. Intervention adherence to PRO-based remote follow-up was exclusively calculated among patients in the intervention groups (N=105). Adherence was defined as the patient being able to complete the questionnaires, measure their blood pressure, and have blood samples taken prior to each of the 6 PRO consultations without assistance from the clinicians or the researcher. The percentage of successfully completed PRO consultations was used as a proxy measure for adherence. An adherence rate of 100% indicated that the patients successfully attended each consultation without assistance from anyone. Engagement in the intervention was measured by calculating as number of questionnaires distributed to patients/number of questionnaire responses received from patients, including the need for sending reminders. The extent of nonresponders was calculated. Furthermore, the item response rate was measured as total number of items distributed to patients/number of item responses. The time spent completing the questionnaire and paper/electronic version distribution was automatically logged in the AmbuFlex database [30].

### Fidelity

Fidelity measured the extent to which the intervention was delivered as planned. Fidelity was measured by counting the number of physicians using the system, including the extent to which the physicians assessed the patients’ responses. Furthermore, the mean number of physicians involved in each patient pathway was reported. The patients’ preferences for mode of contact with their physicians, as well as the extent and nature of the clinical assessment of their responses, was obtained. This information was automatically registered in the AmbuFlex database [30]. A study nurse or the project researcher obtained a quantification of patients who needed course support during the trial and registered in the Research Electronic Data Capture (REDCap) system [31]. Course support was defined as the patient asking for help from the researcher or the project nurse regarding completion of the study procedures. Evaluation components of dose and fidelity were described separately for each PRO intervention group.

### Clinical Engagement

There is no universal definition of clinical engagement; it may be an attitude, a behavior, or an outcome [32]. We defined clinical engagement as the physicians’ perception, attitude, and satisfaction of using PRO actively in the decision-making processes in remote renal care. The clinical engagement toward using PRO-based remote follow-up in clinical practice was explored among the renal physicians who delivered the intervention. We used a qualitative approach inspired by Braun and Clarke’s [33] 6-phase thematic analysis. Individual semistructured interviews with purposively sampled physicians (N=10) most experienced with PRO-based follow-up were performed. The interviews were conducted by the project researcher (author BEG) with experience in qualitative research and occurred immediately after the physicians completed the patients’ final PRO consultation in one of the involved outpatient clinics. An interview guide was used to elicit clinical engagement, asking about the physicians’ views toward the PRO intervention and how they had experienced following the patients remotely (Table S2 in Multimedia Appendix 2). During each interview, notes were written down by BEG, and statements were summed up at the end of each interview and confirmed by the interviewee. These notes constituted the data material. For analysis of all the notes, an analytic coding scheme was developed based on the interview questions and an initial reading of the notes from the interviews (Table S3 in Multimedia Appendix 3) [34]. Citations used in this paper have been translated from Danish.

### Data Analyses

Quantitative data were analyzed using descriptive statistics, such as frequencies, means (SDs), or median (IQRs), as appropriate. Descriptive quantitative information on reach, dose, and fidelity was provided. The presentation of variations between patients in the 2 intervention arms in terms of dose and fidelity were presented. The participation rate was calculated as number of patients consenting to participate/number of eligible patients. Reasons for nonparticipation were synthesized into categories by 2 researchers. We classified patients with low adherence by using the lower quartile. Thus, adherence<83% was the threshold defining low adherence to PRO-based remote follow-up. The adherence threshold was estimated from patients in both PRO intervention arms. Deceased and patients who withdrew due to ending follow-up at the hospital were excluded from the analyses. Participation and adherence data were linked to demographic data collected prebaseline of the trial. Personal and clinical characteristics of high or low adherence were described. Differences were determined using the *χ*^2^ test.

Notes from the semistructured interviews constituted the qualitative data. We performed a thematic analysis to extract important aspects that influenced the physicians’ engagement toward PRO-based remote follow-up. First, all notes were read and re-read several times for familiarization with the data. Second, for an overview of the complete data, statements for each patient were entered into a Microsoft Excel spreadsheet by interview question. Third, preliminary themes that were relevant to the aim of evaluation were identified. Fourth, a coding framework was developed based on the initial themes. Finally, themes were identified and reviewed against the original statements and context. The quantitative and qualitative analyses and reporting were conducted prior to knowing the trial outcomes to avoid biased interpretation of the results [26].

### Ethical Considerations

Patients provided written consent to participate in the trial, including the use of information from their medical records, the AmbuFlex database, and registers. Additionally, verbal consent was obtained from the physicians participating in the interviews. The study was approved by the Danish Authorities for Health Research (number 1-45-70-8-22).

## Results

### Reach

In total, 1060 patients with CKD were screened for participation in the PROKID trial (Figure 1). Of these, 320 (30.2%) patients were found eligible, and 152 (47.5%) agreed to participate in the PRO-based remote follow-up intervention. No statistically significant differences in patient and clinical factors were found between participants and nonparticipants (Table 2). A tendency toward lower participation by older age and lower health literacy was seen. The reasons for not participating in the PRO-based intervention are outlined in Table 3.

**Table 2:** Characteristics of patients with CKD^a^ (N=320) among participants and nonparticipants from Aarhus University Hospital, Gødstrup Hospital, and Viborg Regional Hospital in the PROKID^b^ trial from January 2019 to August 2021.

Characteristics		Total patients (N=320)	Participants (n=152)	Nonparticipants (n=168)
**Age (years), median (IQR)**		74 (12)	74 (11)	75 (11)
	≤69, n (%)	91 (28.4)	48 (31.6)	44 (26.2)
	70-79, n (%)	147 (45.9)	70 (46.1)	76 (45.2)
	≥80, n (%)	82 (25.6)	34 (22.4)	48 (28.6)
**Gender, n (%)**				
	Male	200 (62.5)	98 (64.5)	102 (60.7)
**Renal function**				
	eGFR^c^, mean (SD)	29.2 (6.7)	28.6 (6.2)	29.8 (7.1)
	CKD 3b, n (%)	151 (47.2)	70 (46.1)	81 (48.2)
	CKD 4/5, n (%)	155 (48.4)	82 (53.9)	73 (43.5)
	Missing values, n (%)	14 (4.4)	N/A^d^	14 (8.3)
**Educational level, n (%)**				
	Low (<10 years): none/short <1 year	93 (29.1)	37 (24.3)	56 (33.3)
	Medium (10-12 years): skilled worker/short	148 (46.3)	71 (46.7)	77 (45.8)
	Long (>12 years): middle/long higher	58 (18.1)	29 (19.1)	29 (17.3)
	Missing values	21 (6.6)	15 (9.9)	6 (3.6)
**Labor market affiliation, n (%)**				
	Employed	43 (13.4)	20 (13.2)	26 (15.5)
	Unemployed (retirement, early retirement)	259 (80.9)	119 (78.3)	140 (83.3)
	Missing values	18 (5.6)	13 (8.6)	2 (1.2)
**Comorbidity (Charlson index), n (%)**				
	High (>2)	110 (34.4)	52 (34.2)	58 (34.5)
	Medium (1-2)	195 (60.9)	94 (61.8)	101 (60.1)
	Low (0)	15 (4.7)	6 (3.9)	9 (5.4)
**Health Literacy Questionnaire (HLQ) 4: social support for health, n (%)**				
	Mean (SD)	3.3 (0.55)	3.2 (0.50)	3.2 (0.59)
	Median (IQR)	3.2 (0.6)	3.2 (0.6)	3.2 (1)
	Missing values, n (%)	21 (6.6)	15 (9.9)	6 (3.6)
**HLQ 6: ability to actively engage with health care providers**				
	Mean (SD)	3.9 (0.71)	3.9(0.70)	3.8 (0.71)
	Median (IQR)	4 (0.8)	4 (0.8)	4 (1)
	Missing values, n (%)	21 (6.6)	14 (9.2)	7 (4.2)
**HLQ 9: understanding health information well enough to know what to do**				
	Mean (SD)	3.9 (0.67)	3.9 (0.60)	3.9 (0.72)
	Median (IQR)	4 (0.8)	3.8 (0.8)	4 (0.8)
	Missing values, n (%)	21 (6.6)	14 (9.2)	7 (4.2)
**Self-efficacy (General Self-Efficacy [GSE] scale)**				
	Mean (SD)	29.8 (5.4)	30 (5)	29.6 (5.7)
	Median (IQR)	30 (8)	30 (7)	30 (8)
	Missing values, n (%)	23 (7.2)	14 (9.2)	9 (5.4)
**General health, n (%)**				
	Excellent/very good	53 (16.6)	29 (19.1)	24 (14.3)
	Good	149 (46.6)	63 (41.4)	86 (51.2)
	Fair/poor	114 (35.6)	60 (39.5)	54 (32.1)
	Missing values	4 (1.3)	N/A	4 (2.4)

^a^CKD: chronic kidney disease.

^b^PROKID: PRO measures in Kidney care.

^c^eGFR: estimated glomerular filtration rate.

^d^N/A: not applicable.

**Table 3:** Reasons for nonparticipating in the PRO^a^-based remote follow-up intervention among patients with CKD^b^ (n=168) at Aarhus University Hospital, Gødstrup Hospital, and Viborg Regional Hospital.

Reasons		Nonparticipants (n=168), n (%)
**Patient-reported reasons**		
	Unknown (information unavailable)	17 (10.1)
	Did not want to participate (no further reasons reported)	15 (8.9)
	Could not cope with participating	14 (8.3)
	Preferred telephone consultations/no attendance	13 (7.7)
	Had enough of coping with comorbidity	13 (7.7)
	Visual or hearing disability	11 (6.5)
	Memory issues	7 (4.2)
	Preferred the standard follow-up program	6 (3.6)
	Attends another outpatient follow-up (requiring attendance)	5 (3.0)
	Did not wish to complete the questionnaire	4 (2.4)
**Clinical reported reasons**		
	Health care professional reasons^c^	52 (31.0)
	Other^d^	10 (6.0)

^a^PRO: patient-reported outcome.

^b^CKD: chronic kidney disease.

^c^Ended follow-up, rapid illness progression, compliance issues, comorbidity.

^d^Died before enrollment, departed, other study participation.

### Dose

**Figure 3:**
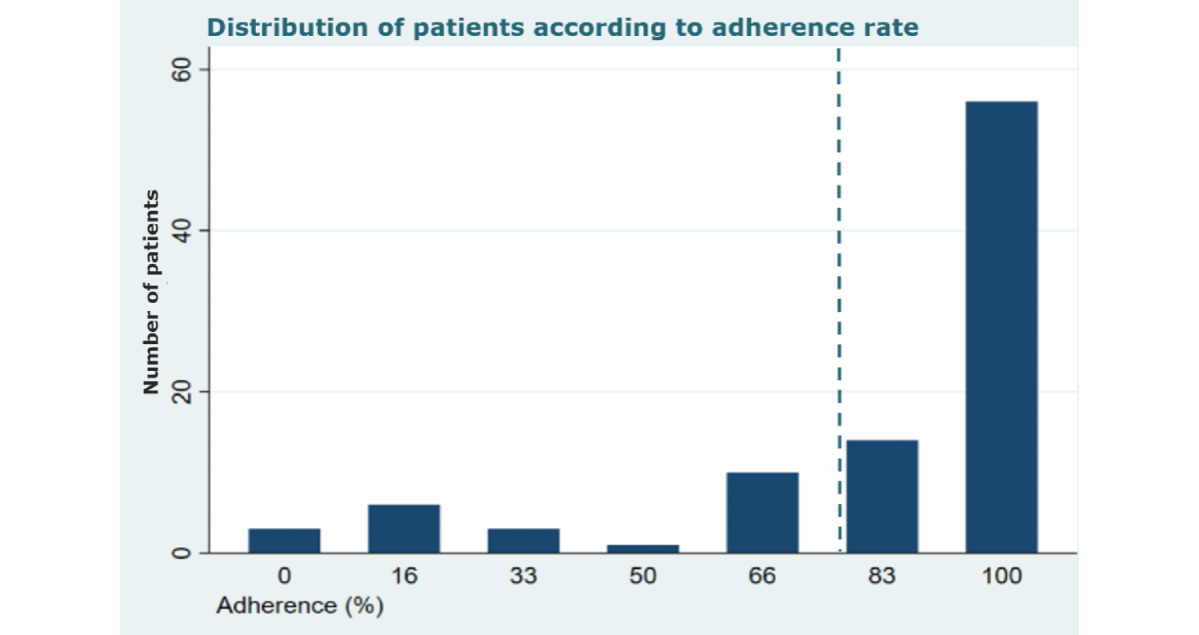
Distribution of patients with CKD in the PRO intervention arms according to adherence to the intervention. Threshold (dotted vertical line) indicating low adherence divided at the lower quartile (<83%) of the distribution (n=93, 88.6%). CKD: chronic kidney disease; PRO: patient-reported outcome; PROKID: PRO measures in Kidney care.

Of the 152 patients, 105 (69.1%) were allocated to the 2 PRO-based intervention arms. Of those, 8 (7.6%) patients died during follow-up and 4 (3.8%) left the study due to termination of outpatient follow-up, leaving 93 (88.6%) patients adhering to PRO-based remote follow-up (Figure 1). The mean adherence rate to the intervention was 82% (95% CI 76-87). In total, 70 (75.3%) of 93 patients had an adherence rate ≥83%, as indicated by the dotted vertical line in Figure 3. The distribution of patients according to the level of adherence rates is shown in Figure 3.

Overall, no statistical differences were found in patient and clinical factors between patients with high or low intervention adherence, albeit a tendency toward lower adherence by poor self-reported health status and lower patient activation was seen (Table S4 in Multimedia Appendix 4). Patients in the PRO-based follow-up group had a significantly lower adherence rate than patients in the PRO-based telephone follow-up group (Table 4).

The overall response rate was 99.1% (539/544). Accordingly, the item response rates were relatively high, with a mean response rate of 96.7% (14,314/14,788; Table 4). In total, 453 (84%) of the 539 patients responded electronically, and they spent a median of 9.22, IQR (6.87) minutes completing the questionnaire.

**Table 4:** Results of the process evaluation of the PROKID^a^ trial among patients with CKD^b^ (N=101) from Aarhus University Hospital, Gødstrup Hospital, and Viborg Regional Hospital.

Elements of process evaluation			Total patients (N=101)		PRO^c^-based follow-up (n=48)		PRO-based telephone follow-up (n=53)
**Dose**							
	Adherence rate, mean % (95% CI)	82 (76-87)		73 (63-83)		90 (84-95)	
	Questionnaire response rate response/total, n/N (%)	539/544 (99.1)		249/250 (99.6)		290/294 (98.6)	
	Item response rate/ total, n/N (%)	14,314/14,788 (96.7)		6329/6696 (95.5)		7985/8092 (98.7)	
	Time completing questionnaire (minutes), median (IQR)	9.22 (6.87)		9.37 (6.97)		9.20 (6.78)	
	Reminders, n/N (%)	367/367 (100)		174/367 (47.4)		193/367 (52.6)	
	Web responses, n/N (%)	453/539 (84.0)		199/249 (89.9)		254/290 (87.6)	
	Assessed questionnaires, n/N (%)	477/539 (88.5)		215/249 (86.3)		262/290 (90.3)	
	Course support^d^, n/N (%)	13/101 (12.9)		9/48 (18.8)		4/53 (7.5)	
	Involved physician per patient, median (range)	3 (1-7)		3 (1-6)		3 (1-7)	
**Color coded algorithm, n/N (%)**							
	Red^e^	N/A^f^		151/249 (60.6)		N/A	
	Yellow^g^	N/A		83/249 (33.3)		N/A	
	Green^h^	N/A		15/249 (6.0)		N/A	
**Clinical assessment**							
	Contact, n/N (%)	417/539 (77.4)		137/249 (55.0)		280/290 (96.6)	
	No contact, n/N (%)	122/539 (22.6)		112/249 (45.0)		10/290 (3.4)	

^a^PROKID: PRO measures in Kidney care.

^b^PRO: patient-reported outcome.

^c^CKD: chronic kidney disease.

^d^The patient asked for help from the researcher or the project nurse regarding completion of study procedures.

^e^High symptom burden or the patient wishes for contact.

^f^N/A: not applicable.

^g^Some symptom burden or the patient may need contact.

^h^No symptom burden and the patient needs no contact.

### Fidelity

The patients completed 539 questionnaires, and in 477 (88.5%) of the 539 cases, a physician assessed their responses (Table 4), leaving 11.5% (62/539) of the questionnaires unnoticed by a physician. The algorithm indicated that 234 (94%) of the 249 patients in the PRO-based follow-up group experienced some disease burden or needed clinical contact flagged by a red or yellow color code. When this was assessed, the physicians found that 137 (55%) of the 249 patients needed to be contacted and 112 (45%) did not need to be contacted. In 288 (69.1%) of all the 417 cases, the physician initiated the contact, and in 129 (30.9%) of the cases, the patient requested contact. The primary causes of contact (242/417, 58%) were attributed to clinical data, including blood samples and blood pressure. Contact based on PRO answers alone accounted for 22.1% (92/417) of the contacts, while concerns related to medication and a precautionary approach prompted the remaining contacts (83/417, 19.9%). In total, 10 (3.4%) of the 290 patients in the PRO-based telephone follow-up group were unavailable when the physician contacted them by phone. On average, 3 (range 1-7) physicians were involved in each patient pathway. A minority of 13 (12.9%) of 101 patients needed assistance to complete study procedures. Common reasons for needing assistance were measuring blood pressure and obtaining information about how to undergo blood tests or how to access the questionnaire. A study nurse or the researcher contacted the patient to address the reasons behind noncompliance and collaboratively devise a plan to ensure future adherence. The majority of those needing assistance were in the PRO-based follow-up group.

### Clinical Engagement

The physicians emphasized that their perception and commitment to the use of PRO-based remote follow-up was influenced by the fact that the PROKID intervention enriched their consultations, as a physician explained:

I believe patients receive a more thorough and attentive consultation through this approach, where all symptoms are carefully examined.

Physician 9

However, PRO-based follow-up also seemed to limit their ability to assess the patients’ health status, as explained in the following by a physician:

I find it quite straightforward to assess individuals over the phone. However, what I miss is observing their movement from the waiting room to the consultation room; it reveals a great deal, such as being able to better evaluate characteristics for instance like skin color and psychical impairment.

Physician 4

On the one hand, all physicians felt that the PROKID intervention is beneficial for their ability to manage and identify patients’ symptoms and estimate their need for contact. The clinicians’ motivation to review the patients’ responses was heightened because the questionnaire included clinical data, such as information about blood pressure and weight.

The clear advantage lies in having everything compiled in this questionnaire, which significantly aids in retaining and managing all the information.

Physician 3

On the other hand, the physicians were also concerned that they might overlook something in the patients’ health status. This 2-sided notion was described in an interview in the following way:

You might lose some connection with the patients, the familiarity that comes with knowing them, and eventually find yourself relying more on questionnaires and blood tests. This shift can make the interaction feel less personal…Conversely, we receive something self-reported (PRO) that delves into deeply personal matters. The risk lies in potentially overlooking something, especially when the patient prefers minimal contact.

Physician 10

To remedy this risk of overlooking symptoms, the physicians wished to combine remote and face-to-face consultations in the future implementation of the PROKID intervention’s remote follow-up. Specifically, the assessment of patients in the PRO-based follow-up group troubled the physicians, as these patients were not automatically contacted by phone as opposed to patients in the PRO-based telephone follow-up group. This was described by a physician in the following way:

I sometimes feel concerned about patients in PRO-based follow-up, who does not visit the hospital. For instance, does this woman truly comprehend the extent of her illness? Is she receiving the necessary information? She may be unaware of what she does not know.

Physician 5

The thematic analysis revealed important aspects that influenced the physicians’ engagement in using PRO. These aspects represented facilitators of and barriers to the implementation of PRO-based remote follow-up in clinical practice. As shown in Textbox 1, the physicians’ engagement in the implementation of PRO-based remote follow-up was supported by several facilitating factors—for instance, PRO enabled an excellent overview of the patients’ health status and increased the possibility for visualization and clarity, as described in this quote:

I wouldn’t have inquired about this if the patient had not communicated it to me. It has completely altered the course of our conversation.

Physician 5

**Physicians’ perceptions on potential facilitators and barriers for establishing clinical engagement in the use of patient-reported outcome (PRO) in remote renal care at Aarhus University Hospital, Gødstrup Hospital, and Viborg Regional Hospital.**

**Facilitators of PRO use in remote care:**

- Better overview of the patient’s overall health condition
- Ensured relevant data are available and lined up
- A great basis for visualizing the patient provided by the combination of PRO and blood tests
- Qualified phone consultations
- Enhanced the patients’ ability to reflect on their symptoms
- Enabled patient-centered focus
- More convenient for the physician and the patient compared to usual follow-up
- An excellent supportive tool for patients during a period of stable illness

**Barriers to using PRO in remote care:**

- Decrease in physicians’ ability to sense the patients’ condition through clinical observation
- Decrease in physicians’ knowledge of patients
- Decrease in crucial knowledge on patient medical history
- Induced risk of forgetting the responsibility of the patient
- Provoked a lower personal relationship with the patient
- Training needed to manage patients’ health status remotely
- Specific patient flow needed to maintain clinical skills of using PRO
- Incentive required by physicians to assess patients’ PRO responses

PRO was also perceived as enabling patient-focused care, to be convenient for both the patient and the clinic, as described by this physician:

It must be convenient for someone like him, who does not have to make the one-hour trip every third month just to let me pressure his angles a bit. We can just as easily handle that over the phone.

Physician 2

However, some barriers to the physicians’ engagement in implementing PRO-based follow-up worked against some of the facilitating factors. Specifically, the physicians faced challenges in accurately evaluating the patients’ health condition and determining their requirement for medical attention and intervention. This was mainly due to the lack of face-to-face interaction and the inability to use their clinical observation skills.

## Discussion

### Principal Findings

In this process evaluation, the intervention processes of PRO-based remote follow-up (the PROKID trial) were evaluated [7]. In total, 47.5% of the eligible patients participated, and they reflected the target population. Overall, we found high percentages of the utility of the system. The dose ranged from 73% to 99%, with a mean adherence rate of 82%. Fidelity showed high usage by physicians, with 88.5% of the total questionnaires being assessed. In total, 12.9% of patients needed assistance to comply with study procedures; this need was predominantly observed in the PRO-based follow-up group. Ten physicians were interviewed about their engagement in the PROKID intervention; most of them rated PRO as being of additional value. However, some barriers, such as decreased knowledge of patients and a lack of using their clinical observational skills, were highlighted.

Overall, the percentages of utility were high in the PRO-based intervention. We believe that several reasons may explain this success. First, an internal pilot study was conducted in an outpatient clinic before trial onset. The pilot study showed that patients were willing to participate and that it was possible to deliver the intervention. We spent time observing the routines and activities in the outpatient clinic to inform our intervention. We had continuous dialogues and meetings with staff members and patients to target the intervention to existing clinical practice. Several studies have highlighted the importance of patients and health care providers knowing why PROs are used and the relevance of the questions asked [35,36]. The fact that the intervention was implemented in a real-life setting and that the users were involved in its development may have impacted patient and clinical engagement with PRO-based remote follow-up [4,37].

Another possible explanation for the high usage percentages may be the procedures used to approach and recruit patients. We included newly referred patients, and it became clear that most preferred no follow-up or remote follow-up, probably due to a feeling of being more ill when entering a hospital [38] or a general lack of experience attending outpatient follow-up. Patient-reported reasons for nonparticipation confirmed this finding, as 7% of the eligible patients declined participation due to preference for telephone consultation or no attendance. Thus, trial participation had the advantage that patients could attend remote follow-up, which was not part of the routine practice at the time. Finally, the degree of involvement of the project researcher and the clinical study nurse in the recruitment phase and during the trial may have motivated patients to participate and made them adhere to and use the intervention. In total, 12.9% of the patients needed course support, highlighting the benefit of having someone to call when problems occur. Prior findings from a parallel qualitative study, in which we interviewed 15 patients in the PROKID trial, support this finding [38].

A well-known concern in RCTs is the challenge of inducing selection bias caused by participation of a selected group failing to represent the target population [24]. We did not find any personal or clinical differences between participants and nonparticipants in our study, which adds value to the generalization of the results of the PROKID trial. Wiegel et al [12] found, in a recently published systematic review, an overall adherence to repetitive electronic PROs in populations with chronic diseases ranging between 61% and 96%, which corresponds well with the mean adherence of 82% found in our study. However, the definition of adherence is known to vary between studies [12]. We believe that having a predefined cutoff point for low adherence may help researchers and clinicians identify patients needing further assistance. Presumably, due to low power, we found no differences in personal or clinical factors between patients with low or high adherence. This might challenge clinicians seeking to identify patients most suitable for PRO-based remote follow-up. The definition and threshold for adherence correspond to the goal of using remote PRO in the outpatient clinic, as in the real-life setting, patients should be capable of completing the PRO questionnaire by themselves. We found a high response rate in our study, contrary to other previous findings [35]. We believe the 2 main reasons for this were the high use of reminders and the fact that a study nurse called the patient if questionnaire responses had not been submitted at the date of the PRO consultations. The use of reminders is known to increase the response rate [39]. Knowledge of the extent of this phenomenon is important to convey to the outpatient clinics when the intervention is widely implemented.

Another interesting finding is the number of physicians involved in each patient pathway. Preferably, patients should have 1 contact physician during follow-up, but we saw that each patient had contact with 3 (range 1-7) physicians during the 18 months’ trial. Evidence from a meta-analysis suggests that for the safety and continuity of care, it is essential to have as few different health care professionals as possible [40]. A qualitative study integral to the PROKID intervention concluded that barriers to patient engagement in PRO-based remote follow-up are unfamiliarity with the physician and remote follow-up challenging the patient-physician relationship [38]. Furthermore, the involvement of multiple physicians in reviewing each patient over the 18 months posed a challenge, leading to diminishing familiarity with the medical history of the patients. Remote monitoring is increasingly substituting traditional hospital visits [41]. A recent study argued that telephone follow-up is as safe as visits to the outpatient clinic, especially if the patient is acquainted with the physician [42]. Thus, it seems crucial to incorporate individual patient-physician consultations to the extent possible.

A prior study of field observations during the PRO consultations showed that the extent to which the physicians incorporated the patient responses into the consultations reached almost 100% [38]. However, data from this process evaluation study found that the physicians merely assessed 88.5% of the questionnaire responses received. This might indicate observer bias in the qualitative study [38], showing a falsely high result, although it might also indicate a false lower result in our study because of recall bias, as it seemed as if the physicians actually opened and assessed the patients’ PRO responses but forgot to approve and document the responses in the AmbuFlex database. However, it is a well-known challenge to get clinicians to assess and approve patients’ PRO responses [19,43]. The qualitative analyses showed that the physicians found PRO-based remote follow-up to enrich the consultations, but it also induced a risk of failing to notice important symptoms. Prior research has shown that engagement decreases when activities or tasks are perceived as wasteful of resources or creating more harm than benefit [44]. Therefore, enhancing the physicians’ satisfaction and perception of the usability of implementing PROs in remote care seems crucial. The physicians emphasized that they needed an incentive to open the patients’ PRO responses. During the interviews, it became clear that the patients’ blood pressure and weight were the physicians’ primary objects of interest. This result was supported in a qualitative study on patient perspectives [38] and has also been found to be an issue in other studies [22,45]. Hence, an important factor driving the assessment of patients’ PRO responses was the incorporation of the self-reported clinical outcomes (blood pressure and weight) in the questionnaire.

The color-coded algorithm showed that most of the patients needed contact with a physician or were burdened by disease-specific symptoms. Thus, the proportion of patients contacted by physicians was relatively high, which may indicate a high claim for clinical attention in this patient group. However, it may also indicate a sensitive algorithm [4]. The clinical experts aimed to develop an algorithm with high sensitivity since a low level of false-negative cases was more important than a high level of false-positive cases. Thus, the physicians’ ability to assess whether to contact patients was pivotal for the follow-up process. Further studies that consider the results of the green-yellow-red color-coded algorithm will need to be undertaken.

### Strengths and Limitations

Process evaluation was considered integral to the PROKID trial, and the results of this process evaluation were analyzed prior to knowledge of the trial outcomes. Thereby, the risk of bias was reduced [46]. This study enabled us to perform a more detailed examination of the process of recruitment and intervention adherence to inform the interpretation of trial results and generalizability [47]. A main strength of this study is that it included patients from a well-defined population covering all centers following patients with CKD in Central Denmark Region. Quantitative and qualitative methods complemented one another, and a combination of self-reported, qualitative data and register-based data was used to link different data sources. Another key strength of this study was the use of Steckler and Linnan’s [26] process evaluation framework, which allowed us to structure the process evaluation appropriately. Observations and interviews had the potential to influence the involved physicians’ and patients’ behavior and commitment. However, we believe that the timing of the evaluation in the finishing part of the trial limited this effect. The evaluator responsible was also the principal investigator in the PROKID trial, which provided insight into and understanding of the context, as an outsider could not have added to the findings.

This study also has some limitations. Initial development and use of a logic model may have better illustrated the underpinning theory [26]. The absence of predefined core questions was also a notable limitation. In the qualitative data analyses, the researcher’s familiarity with the participating departments may have induced a certain risk of bias. This may have led to a more positive attitude toward PRO-based remote follow-up among the physicians. However, it may also have made them confident that critical comments would be received constructively.

### Conclusion

The PRO-based PROKID intervention was generally well received by patients and physicians, as we found high percentages of utility across all process evaluation components. Our findings highlight that remote PRO-based follow-up is feasible in a clinical setting and may be a relevant and valuable tool when remotely monitoring patients with CKD. We found high adherence and response rates among the patients, and the physicians assessed the majority of patient responses. However, it seems important to have a key figure who helps patients in need of assistance to comply with PRO-based remote follow-up. The physicians emphasized both enabling factors, such as improved overview and patient-focused care, and barriers, particularly the complexities of evaluating health conditions without direct interaction. Suggestions for future implementations included a combination of remote and face-to-face consultations to address this concern.

## Appendix

Multimedia Appendix 1 Investigated patient and clinical factors.

Multimedia Appendix 2 Interview guide.

Multimedia Appendix 3 Preliminary coding scheme aiming to explore the physicians' perceptions and experiences using PRO-based remote follow-up. PRO: patient-reported outcome.

Multimedia Appendix 4 Characteristics of patients adhering to PRO-based follow-up according to demographic, disease-related, and individual determinants divided into high (≥83%) and low (<83%) adherence (n=93). PRO: patient-reported outcome.

Multimedia Appendix 5 CONSORT-EHEALTH checklist.

## Abbreviations

CKD: chronic kidney disease
eGFR: estimated glomerular filtration rate
PRO: patient-reported outcome
PROKID: PRO measures in Kidney care
RCT: randomized controlled trial
REDCap: Research Electronic Data Capture

## Data Availability

The data sets generated and analyzed during this study are available in anonymous form from the corresponding author on request or by contacting the AmbuFlex – Centre for Patient-reported Outcomes.
