# Supplementary material for: Remote Symptom Monitoring Using Patient-Reported Outcomes in Patients With Chronic Kidney Disease: Process Evaluation of a Randomized Controlled Trial
Source: JMIR Form Res. 2024 Apr 24;8:e48173. doi: 10.2196/48173 (PMC11079764; doi:10.2196/48173)
Supplement: Multimedia Appendix 1 [file formative_v8i1e48173_app1.docx]

Supplemental Table 1. Investigated patient and clinical factors

| **Variables** | **Type of variable** | **Data sources** |
| --- | --- | --- |
| **Patient factors** |  |  |
| Age | Continuous | The Hospital Business Intelligence Register (Central Denmark Region) |
| Gender | Categorical | The Hospital Business Intelligence Register (Central Denmark Region) |
| Education | Categorical  Low (<10 years) Medium (10-12 years)  Long (> 12 years) | Questionnaire data |
| Labour market affiliation | Dichotomized  Employed/non-employed | Questionnaire data |
| Self-efficacy | 0-40 (best) | General Self-Efficacy Scale (GSES) |
| Health literacy | 1 (worst) to 5 | Health Literacy Questionnaire (HLQ), sub scale 4, 6 and 9 |
| Patient activation | 1 (worst) to 4 | Patient Activation Measure (PAM) |
| General health | 1-5 (best) | Short-form 36 (SF-36) |
| Reasons for not participating | Categorical | Patient or clinician reported |
| **Clinical factors** |  |  |
| Comorbidity | Categorical  0 (Low),  1-2 (Medium)  >2 (High) | Charlsons Comorbidity Index The Hospital Business Intelligence Register (Central Denmark Region) |
| Renal function | Estimated glomerular filtration rate (egfr)  Continuous | The Hospital Business Intelligence Register (Central Denmark Region) |
