# Supplementary material for: Remote Symptom Monitoring Using Patient-Reported Outcomes in Patients With Chronic Kidney Disease: Process Evaluation of a Randomized Controlled Trial
Source: JMIR Form Res. 2024 Apr 24;8:e48173. doi: 10.2196/48173 (PMC11079764; doi:10.2196/48173)
Supplement: Multimedia Appendix 2 [file formative_v8i1e48173_app2.docx]

### Interviewguide: physicians perceptions

| INTRODUCTION | |
| --- | --- |
| Date:  Informant:  Woman: □ Man: □  Place for interview:  Age: | |
| **THEME** | **QUESTIONS** |
| **Preliminary questions** | For how long have you been a medical doctor? |
|  | What is your present position?  □ Junior doctor  □ Consultant  □ Senior consultant  □ Other: |
|  | For how long have you worked with patients with chronic kidney disease? |
|  | How long have you been employed at this department? (Number of months/year) |
|  | How experienced are you in working with PRO-data? (Years/months) |
|  | For how long have you been a part of the PROKID intervention (Years/Months) |
|  | How often do you use the PRO system?  □ Daily or several times a week  □ Weekly  □ Monthly  □ Rarely  □ Never |
|  | |
| **Structurel questions** | Do you think PRO is an additional help for you?  Why/why not?  Which benefits do you see for the patients using this system?  Is PRO-data compatible with the way you structure your workflow in the outpatient clinic?  In what way do you think, the patient had influence on the conversation when using PRO  What determines whether PRO is used in the outpatient clinic? |
| **Assessment of a PRO response**  **(observation)** | Are some symptoms more important for you than others?  - Which and why?  Do you tend to compare the current response with previous responses?  - why/why not? |
| **Use of data from the electronic medical record**  **(+observation)** | Do you compare data from eg. the medical journal with the questionnaire response?  - What does this data contribute to? |
| **Clinical decision-making**  **(+ observation)** | How do you visualize the patient when you assess the PRO response?  How do you perceive the fact that you don't sit face-to-face with your patient during the consultations?  Do you often have issues relating to PRO, that you need to clarify?  How did you make a plan for the further care plan for the patient? |
| **Clinical tasks** | How do you make sure all tasks are taken care of after the consultation?  How do you collaborate with the nurses? |
|  | |
| **Overall perception of the PROKID intervention and its effect on the quality of care in the outpatient clinic** | How do you think the patients experience being followed remotely?  What do you think the patients gain from completing the disease-specific questionnaires?  What significance/impact have the implementation of PRO had for the outpatient follow-up in the clinic?  Can this (PROKID intervention) be continued as an offer in the outpatient clinic? Should it be altered?  If yes, which alterations should be done? |
|  | |
| **Workflow**  **Implementation** | Which factors must be present for the intervention to be wider implemented in clinical practice?  Has your workflow changed after implementing PRO?  How have you been informed and trained in using the PRO-system |
|  | |
| **Final remarks**  **Summary** | In summary, what I have heard you say is…(content)  Is that correctly understood?  Do you have comments for this?  If you have further to ad, do not hesitate to call or write me an email. |
