# Supplementary material for: Remote Symptom Monitoring Using Patient-Reported Outcomes in Patients With Chronic Kidney Disease: Process Evaluation of a Randomized Controlled Trial
Source: JMIR Form Res. 2024 Apr 24;8:e48173. doi: 10.2196/48173 (PMC11079764; doi:10.2196/48173)
Supplement: Multimedia Appendix 3 [file formative_v8i1e48173_app3.pdf]

Supplemental Information 3.

Preliminary coding scheme aiming to explore the physicians' perceptions and experiences using PRO-based remote follow-up.

**Preliminary themes**	**Quotations**
PRO constitutes an overview	*"PRO is a great help when you have a telephone consultation. Here, you get all the necessary information, e.g. blood pressure and weight. You can also see how the patient is doing compared to before. This is really an advantage of this questionnaire."* (PH 13)<br>*" Telephone consultations have been upgraded using the questionnaires"* (PH 4)<br>"*It has become much easier to proceed systematically when you have this list of symptoms*" (PH 4)<br>*"I think it gives a good overview when assessing the patient. It is quick to look down at the answers and see which symptoms the patient is suffering from."* (PH 5)<br>*" it is the history (repeated questionnaires) that makes it all clear".* (PH 10)<br>*"You can expedite the process by addressing the issues more promptly. It is quicker to understand what the patient perceives as a problem. This approach has facilitated my ability to gather crucial information. It is akin to individuals having their medication list pre-filled, enabling more efficient use of time for discussing vital matters."* (PH 10)<br>*"It qualifies my telephone consultation" and saves time, as I can quickly see what we have to talk about. The conversation has less risk of becoming unstructured"* (PH 2)
Remote assessment of the patient's health status	*" We must be aware that home monitoring does not remove us from responsibility for the patient"* (PH 3)<br><br>*"I find it quite straightforward to assess individuals over the phone. However, what I miss is observing their movement from the waiting room to here; it reveals a great deal, such as being able to better evaluate characteristics like skin colour, for instance, detecting if there is a complete lack of colour"* (PH 4)<br><br>*"We receive something self-reported that delves into deeply personal matters. The risk lies in potentially overlooking it, especially when the patient prefers minimal contact."* (PH 10)<br><br>"*It (PRO) qualifies my telephone conversation; we are more systematically asking about symptoms. However, the conversations are more challenging to manage when you do not have the patient in front of you".* (PH 4)<br>*"Sometimes, I lack the clinical look. For example, if this patient has gained 3 kg. He says he does not have water in his legs, but I do not know..."* (PH 2)

Supplemental Information 3.
Preliminary coding scheme aiming to explore the physicians' perceptions and experiences using PRO-based remote follow-up.

Convenience for the patients/ Flexibility	*"In any case, it must be convenient for, for example, him here, who does not have to make the one-hour trip every third month just to let me make a pressure on his ankles - we can just as easily take that over the phone."* (PH 2)<br>*"PRO provides increased flexibility, and it is easier for patients not to enter the hospital every time."*<br>*" An appealing option for patients with a stable condition"* (PH 3)<br>*"I think completing PRO is a help for the patients. It gives them time to think and reflect on their disease before the consultation."* (PH 6)<br>*" It must also save the patients much time. Think of spending 1-1½ hours driving down here to talk to me for 7-10 minutes."* (PH 8)
Remote consultations	*"…if medication changes are to be made, e.g. a change in diuretics, it is easier to have people facing you. However, with CORONA and the strike, we have become more used to working with telephone consultations and can see the value in this".* (PH 2)<br><br>*" Home monitoring with PRO is a useful tool for stable patients, but when they are about to reach the dialysis limit, we will have to see them in the clinic."* (PH 9)<br><br>*"Remote PRO works effectively for me when I am already familiar with the patient. In this case, as I am not acquainted with the lady, I am uncertain and therefore cautious, especially regarding medication adjustments."* (PH 14)<br><br>*" Before the patient even closes the door, I have nearly determined the course of action. Over the phone, I must engage in conversation with them, attentively listening to their reported symptoms. Occasionally, their descriptions of symptoms do not align with what we observe here (in the medical record)."* (PH 7)<br><br>*" I think it's difficult when you don't have the patient in front of you. I have difficulty remembering the patients and that is a problem"* (PH 14).
PRO in the consultation	*"I believe patients receive a more thorough and attentive consultation through this approach, where all symptoms are carefully examined."* *(*PH 9)<br>*"Overall, this is super smart!, especially with telephone consultations. However, you have to remember that it is a support tool. It is important to check blood tests and history thoroughly as well."* (N 14)

Supplemental Information 3.

Preliminary coding scheme aiming to explore the physicians' perceptions and experiences using PRO-based remote follow-up.

Patient-centred approach	*"I would not have inquired about this if the patient had not communicated it to me. It has completely altered the course of our conversation."* (PH 5)<br>*"I appreciate it when patients provide their own conversation topics. In response to questions, the patient simply provides an answer that needs to be checked off. The conversation topics are where the patient has influence, and we talk real patient involvement."* (PH 8)
Using PRO in the clinical encounter	*"We simply need to utilize it more frequently. The more we use it, the more effective it becomes as a tool, reinforcing our habit of incorporating it".* (PH 6)<br>*"In consultations, there is a lot to remember – from checking blood tests and blood pressure to examining urine and now incorporating PRO. The clear advantage lies in having everything compiled in this questionnaire, which significantly aids in retaining and managing all the information."* (PH 3)<br><br>*"Opening all those systems within a 10-minute patient window is challenging. Navigating through the extensive information the patient provides can be difficult, considering the complexity of the scheme. Additionally, understanding the patient's expectations for questionnaire completion is crucial, and I do not always utilize all aspects of the system."* (PH 9)
Relation/connection with the patient	*"You might lose some connection with the patients and the familiarity of knowing them and eventually rely more on questionnaires and blood tests. This shift can make the interaction feel less personal. The intriguing aspect lies in understanding whether this barrier holds significance.* (PH 10)<br><br>*" Consistency is important. It might be beneficial if the same physician sees the patient every time. In the first instance, the patient's response is "red"; you have to ask the patient and make him elaborate. After that, you will understand the issue within this patient."* (PH 10)<br><br>*" The relationship can be an advantage and a disadvantage. People may be more open when facing the doctor, but others may feel the opposite."* (PH 10)<br><br>*"PRO is a valuable supplementary tool during consultations; it helps to remember the patient. When taking over another physician's program, assessing the patient's contact needs becomes challenging due to a lack of familiarity with the individual."* (PH 14)

Supplemental Information 3.

Preliminary coding scheme aiming to explore the physicians' perceptions and experiences using PRO-based remote follow-up.

Patients understanding of their disease	*"It would be best if you did not pull this down on their heads (Remote PRO consultations). When there are uncertainties about disease comprehension, it can pose an issue if individuals do not visit the department."* (PH 10)<br><br>*"I sometimes feel concerned about those (patients in PRO-based follow-up) who do not visit the hospital. Does this woman truly comprehend, for instance, the extent of her illness? Is she receiving the necessary information? She may be unaware of what she does not know."* (PH 5)
