# Supplementary material for: Remote Symptom Monitoring Using Patient-Reported Outcomes in Patients With Chronic Kidney Disease: Process Evaluation of a Randomized Controlled Trial
Source: JMIR Form Res. 2024 Apr 24;8:e48173. doi: 10.2196/48173 (PMC11079764; doi:10.2196/48173)
Supplement: Multimedia Appendix 4 [file formative_v8i1e48173_app4.pdf]

Characteristics of patients adhering to PRO-based follow-up according to demographic, disease-related and individual determinants divided to high (≥83 %) and low (< 83 %) adherence. N = 93

	Low adherence, N = 23 n (%)		High adherence, N = 70 n (%)		P value X^2 test
**Age, years**					0.18
≤ 69	7	(30)	23	(33)	
70-79	8	(35)	35	(50)	
≥ 80	8	(35)	12	(17)	
**Gender**					0.86
Male	14	(61)	44	(63)	
**Education**					0.48
High (> 12 years)	6	(26)	12	(17)	
Medium (10-12 years)	10	(44)	34	(49)	
Low (< 10 years)	4	(17)	19	(27)	
Missing	3	(13)			
**Labour market affiliation**					0.79
Employed	3	(13)	8	(11)	
Non employed	18	(78)	58	(83)	
Missing	2	(9)	4	(6)	
**Renal function**					0.53
CKD 3b	10	(43)	31	(44)	
CKD 4/5	13	(57)	39	(56)	
**Comorbidity (Charlson Index)**					0.17
Low 0	-	-	5	(7)	
Medium 1-2	18	(78)	42	(60)	
High > 2	5	(22)	23	(33)	
**General health**					0.14
Excellent/very good	2	(9)	19	(27)	
Good	12	(52)	27	(39)	
Fair/poor health	9	(39)	24	(34)	
**Self-efficacy (GSE)**					0.58
Mean (SD)	30.1	(5.11)	30.3	(5.0)	
Median [IQR]	31	[8]	30	[7]	
**HLQ 4: Social support for health**					0.73
Mean (SD)	3.19	(0.65)	3.22	(0.46)	
Median [IQR]	3.3	[0.8]	3.2	[0.6]	
Missing	2	(9)	4	(6)	
**HLQ 6: Ability to actively engage with healthcare providers**					0.50
Mean (SD)	4.05	(0.64)	3.92	(0.70)	
Median [IQR]	4	[0.8]	4	[0.8]	
Missing	2	(9)	4	(6)	
**HLQ 9: Understanding health information well enough to know what to do**					0.65
Mean (SD)	3.95	(0.59)	3.93	(0.58)	
Median [IQR]	3.8	[0.8]	4	[0.6]	
Missing	2	(9)	4	(6)	
**Patient activation[a)]**					0.21
Agree/strongly agree	19	(82)	64	(77)	
Disagree/strongly disagree	2	(9)	2	(50)	
Missing	2	(9)			
**Patient activation[b)]**					0.21
Agree/strongly agree	17	(74)	60	(86)	
Disagree/strongly disagree	4	(17)	6	(8)	
Missing	2	(9)	4	(6)	

CKD: Chronic Kidney Disease; HLQ: Health Literacy Questionnaire; GSE: General Self-efficacy scale;

[a] Taking an active role in my own health care is the most important thing that affects my health

[b] I am confident I can figure out solutions when new situations or problems arise with my health condition
